# Supplementary material for: Pheromone Mediated Sexual Reproduction of Pennate Diatom Cylindrotheca closterium
Source: J Chem Ecol. 2021 Apr 29;47(6):504–12. doi: 10.1007/s10886-021-01277-8 (PMC8217010; doi:10.1007/s10886-021-01277-8)
Supplement: Supplementary file 1 — Supplementary file1 (DOCX 107 KB) [file 10886_2021_1277_MOESM1_ESM.docx]

Pheromone Mediated Sexual Reproduction of Pennate Diatom *Cylindrotheca closterium*

Franziska Klapper^1^, Sien Audoor^2^, Wim Vyverman^2^, Georg Pohnert^1,3*^

** Georg Pohnert
Georg.Pohnert@uni–jena.de*

*1 Institute for Inorganic and Analytical Chemistry, Bioorganic Analytics, Friedrich Schiller University Jena, Lessingstrasse 8, D-07743 Jena, Germany*

*2 Laboratory of Protistology and Aquatic Ecology, Department of Biology, University Gent, Krijgslaan 281 S8, 9000 Gent, Belgium*

*3 Max Planck Institute for Chemical Ecology, Hans-Knöll-Straße 8, D-07745 Jena, Germany*

**Abstract –** Benthic diatoms dominate primary production in marine subtidal and intertidal environments. Their extraordinary species diversity and ecological success is thought to be linked with their predominantly heterothallic sexual reproduction. Little is known about pheromone involvement during mating of pennate diatoms. Here we describe pheromone guided mating in the coastal raphid diatom *Cylindrotheca closterium*. We show that the two mating types (mt^+^ and mt^¯^) have distinct functions. Similar to other benthic diatoms, mt^+^ cells are searching for the mt^¯^ cells to pair. To enhance mating efficiency mt^¯^ exudes an attraction pheromone which we proved by establishing a novel capillary assay. Further, two more pheromones produced by mt^¯^ promote the sexual events. One arrests the cell cycle progression of mt^+^ while the other induces gametogenesis of mt^+^. We suggest that *C. closterium* shares a functionally similar pheromone system with other pennate diatoms like S*eminavis robusta* and *Pseudostaurosira trainorii* which synchronize sexual events and mate attraction. Remarkably, we found no evidence of mt^+^ producing pheromones, which differentiates *C. closterium* from other pennates and suggests a less complex pheromone system in *C. closterium*.

**Key Words –** Mating Types, *Cylindrotheca closterium*, pennate diatoms, pheromones, attraction assay, cell cycle regulation.

Acknowledgments

The research leading to the results presented in this publication was partly carried out with infrastructure funded by EMBRC Belgium—FWO project GOH3817N and funding from the research council of Ghent University (BOF/GOA No. 01G01715). The authors are thankful for the provision of diatom strains by the BCCM/DCG culture collection. Further, the authors acknowledge the Cell Cycle Group at VIB-UGent Center for Plant Systems Biology for flow cytometer measurements. The authors are grateful to M. Vallet for her suggestions on the improvement of experimental set ups. R. X. Poulin´s help with the design of the figures and his comments on an earlier version of the manuscript are highly appreciated.

Introduction

Diatoms constitute a diverse group of photosynthetic unicellular microalgae and are key players in carbon cycling and nutrient exchange in marine and freshwater ecosystems (Armbrust 2009; Benoiston et al. 2017; Falkowski et al. 1998; Malviya et al. 2016; Smetacek 1998). Benthic raphid pennate diatoms are less studied than the predominantly planktonic group of centric diatoms. Yet, benthic diatoms contribute significantly to primary production in estuaries and coastal regions (Stal et al. 2019; Yallop et al. 1994) where they frequently dominate biofilms and stabilize sediments. Recent genomic studies revealed their unique adaptations to this highly dynamic and heterogeneous environment (Osuna-Cruz et al. 2020). They show an extraordinarily high species diversity, thought to be driven by the evolution of motility and the transition to a heterothallism (Nakov et al. 2018).

Reproduction systems of raphid pennate diatoms have been intensively studied in the last decades (Amato 2010; Chepurnov et al. 2004; Poulíčková and Mann 2019). Asexual division prevails during the diplontic life cycle of diatoms and is accompanied by a gradual cell size reduction. Meiosis and subsequent sexual reproduction, however, are vital to restore original cell size. Typically, sexual reproduction is triggered when cells reach a species-specific sexual size threshold (SST, ~50% of their original size) (Chepurnov et al. 2002; Davidovich 2001), following pair formation between sexually compatible mating types (hereafter referred to as mt: cells  < SST, and as MT: cells  > SST). Although mating types are morphologically indistinguishable, mating type determination has a genetic basis (Vanstechelman et al. 2013, Russo et al. 2018) and is reflected in behavioural and physiological differentiation. For example, in *Seminavis robusta,* mt^¯^ cells produce the attraction pheromone diproline, guiding the movement of the more motile mt^+^ cells towards them (Chepurnov et al. 2005; Chepurnov et al. 2002).
Mate finding and sexual reproduction are energetically costly (Lewis 1987) and pheromones can increase mating probability and efficiency (Basu et al. 2017; Frenkel et al. 2014). In the planktonic diatom *P. multistriata* a pheromone is suggested to synchronize the sexual events (Scalco et al. 2014) whereas three different consecutively induced pheromones are thought to be involved in the reproduction of the freshwater diatom *Pseudostaurosira trainorii* to control sexualization of cells and orientate gametes (Sato et al. 2011). Mating of *S. robusta* was studied intensively and is also promoted by three distinct pheromones (Bilcke et al. 2020; Bondoc et al. 2019; Bondoc et al. 2016; Chepurnov et al. 2002). Two sex inducing pheromones SIP^+^ and SIP^-^ are produced by mt^+^ and mt^¯^, respectively, and synchronize the cell cycle (Moeys et al. 2016). Pheromone structures are poorly studied (Bonneure et al. 2021) but the chemoattractant of *S. robusta* was identified as cyclo(l‑Pro‑l‑Pro) (l‑diproline), the first elucidated attraction pheromone of a diatom (Gillard et al. 2013). The mt^¯^ cells that are exposed to SIP^+^ release l‑diproline to attract mt^+^ cells.

The pennate diatom *Cylindrotheca closterium* (Ehrenberg) Reimann & J.C.Lewin is a meroplanktonic species, exploiting both planktonic and benthic habitats and can be abundant in water columns as well as biofilms. As it is found in marine, brackish, freshwater regions and even inside sea-ice (von Quillfeldt et al. 2003), *C. closterium* has been used as model in several eco-physiological studies showing its ability to adapt to varying temperatures (Stock et al. 2019) and salinities (Najdek et al. 2005). The unique movement modalities of *C. closterium* also change in response to hypo- or hyper-saline conditions (Apoya-Horton et al. 2006). In recent years even a transformation protocol has been established for *C. closterium* and a gene editing protocol through CRISPR/Cas9 is currently developed, thus providing a toolbox for many molecular studies as well (unpublished data).

The life cycle of *C. closterium* has also been studied in detail (Vanormelingen et al. 2013). When cells reach the SST (~70% of their original size) and partners of the two distinct mating types, Cyc1 and Cyc2 meet, sexual reproduction takes place resultin in two cells with the initial cell size. The purpose of this study was to specify the mating behaviour of each mating type and investigate whether there is an attracted (mt^+^) and an attracting (mt^¯^) partner. Further, we aimed to determine if a directed attraction during mate finding occurs. The possibility of pheromone involvement during mating in *C. closterium* was also investigated and similarities and disparities in the mating systems of pennate benthic diatoms are discussed.

Methods and Material

*Strains, Culture Conditions and Microscopy*

C2 (DCG 0977, Cyc1, identified as mt^+^ (see below)) and CA1.15 (DCG 0923, Cyc2, identified as mt^¯^ (see below)) strains of *Cylindrotheca closterium*, both sexually mature (cell size: Cyc1 < 50 µm, Cyc2 < 30 µm) and CZ1 (MT^¯^ > 60 µm; >SST) were provided by the BCCM/DCG diatom culture collection at Ghent University (http://bccm.belspo.be/about-us/bccm-dcg). Cultures were grown in f/2 + Si medium (Guillard 1975) in a 12:12 h light:dark regime at 18 °C with fluorescent lamps at ~30 µmol photons m^-2^ s^-1^. Stocks were kept in T-25 tissue culture flasks (10 mL, Sarstedt, Nümbrecht, Germany) and reinoculated every week to keep them in exponential growth.
To estimate cell density by microscopically counting and surveying experimental results, photographs of each culture (n = 3) were taken with a Nikon DS-Fi2 CCD camera (Tokyo, Japan) attached to an inverted Leica DM IL LED light microscope (Heerbrugg, Switzerland) (100 x magnification). The open-source software ImageJ (Rasband WS, ImageJ, U. S. National Institutes of Health, Bethesda, Maryland, USA. http://imagej.nih.gov/ij/ 1997) and the cell counter plug-in was used for counting cells as well as pairs and clusters of cells in mating experiments.
For experiments cultures were first grown in 6-well plates (5 mL well^-1^, Sarstedt, Nümbrecht, Germany) for 4 days to reach early exponential phase. Thereafter cultures were dark‑synchronized for 36 hrs (Vanormelingen et al. 2013).

*Clustering Experiment*

For mating type designation, dark arrested cultures of compatible strains were crossed in triplicate in 5 different density ratios, 1:9, 3:7, 5:5, 7:3 and 9:1 in 24 well plates, having monoclonal cultures as control. Each well contained 500 µL of spent medium of each strain and 200 µL of cell suspension (30·10^3^ cells cm^-2^) composed of the above mentioned mating type proportions. Five hours after re‑illumination the mating response was evaluated by manually analysing microscopic photographs. Cells of the two mating types were distinguished by the differing apical cell length. The following classes of interacting cells were discriminated: mating-pairs (two cells of different mating type laying against each other with their long axes parallel), multiple pairing cluster (accumulation of cells in which the mating type of the majority is three times more present than the other), as well as the total number of interactions (pairs, clusters and triplets).

*Capillary Assay*

For the investigation of cell attraction, dark‑synchronized cultures of mt^+^ were split into 12‑well plates in new f/2 medium and kept at light for 6 hours.
Two hundred millilitres of cell free medium of exponentially growing cultures of C2, CA1.15 and CZ1 (mt^+^, mt^¯^ and MT^¯^ (>SST) respectively) alike were extracted using HLB cartridges (hydrophilic-lipophilic balanced solid phase extraction, Oasis®, Waters, Eschborn, Germany) following the instructor’s manual. MeOH extracts were dried under nitrogen flow and dissolved in 200 µL pure water (LC/MS grade).
Capillaries (l = 30 mm, V = 5 µL, minicaps, Hirschmann, Germany) were prepared directly before use (n = 4). Therefore, 25 µL of medium extract were added (1:1 v/v) to hot agar (2% Agar-Agar, Kobe 1, Roth, Germany), mixed quickly and immediately absorbed into the capillaries by capillary forces. The outside of the capillaries was cleaned carefully and the capillaries were air-dried for 5 minutes. Capillaries were then carefully placed vertically in the wells which were covered by a plastic foil holding the capillaries in place.
Pictures of the capillary openings were taken at t = 0 min (directly after inserting the capillary), t = 30 min, and t = 60 min and cells were counted within a defined area of d = 580 µm around the capillary (ImageJ).

*Motility Assay*

In 24-well-plates, dark arrested cultures of C2 (mt^+^) and CA1.15 (mt^¯^) were treated with 1.5 µL of medium extract of the opposite mating type (n = 4) (see *Capillary Assay* for extract preparation) four hours after re-illumination. Control cultures stayed untreated. In C2 experiments three individual videos (30 s) were recorded (1fps) for each well one hour after the treatment to verify the robustness of the assay. In experiments with CA1.15 one video was recorded for each replicate. Cell tracking was done for >300 cell per replicate using the plug-in TrackMate (<http://fiji.sc/TrackMate>) for ImageJ.

*G1 Cell Cycle Arrest Assay*

Five mL of medium of exponentially growing cultures of C2 and CA1.15 (mt^+^ and mt^¯^) were sterile filtered (Filtropur S 0.2 µm, Sarstedt, Germany) immediately before the experiment. Dark-arrested cultures were split for different treatments 2 hours before re‑illumination. In the dark, culture medium of treatments was fully exchanged by filtered medium of the opposite mating type while control cultures stayed untreated.

At t = 0 hrs (in the dark) and t = 6 hrs (after re-illumination), 2 mL samples were taken and directly centrifuged (5 min, 3000 rpm). The supernatants were discarded and directly replaced by ice-cold 70% ethanol. These fixed samples were stored at 4 °C in the dark for at least 24h. The supernatant was discarded and cell pellets were resuspended in 200 µL 70% ethanol and washed twice with phosphate-buffered saline (PBS, 137 mm NaCl, 2.7 mm KCl, 10 mm phosphate, pH 7.4). Samples were treated with 1 µg mL^-1^ RNAse A for 40 min at 37 °C and afterwards stained with SybrGold (10,000 fold diluted from stock solution, SYBR Gold Nucleic Acid Gel Stain, Thermo Fisher Scientific, USA) for 10 min in the dark. Cells were transferred to PBS again and filtered over Celltric filters (Sarstedt, USA) to exclude cell clusters. DNA content was measured on a BC Accuri C6 flow cytometer. For each sample, 5000 events were collected and gated in the FL1 and FSC channel. G1 and G2+M peaks were visually selected and statistically analysed.

*Gametogenesis*

Gametogenesis was induced by crossing dark arrested cultures of mt^+^ and mt^¯^ (n = 3) in 24 well plates in either 1:9 or 9:1 ratio. Each well contained 500 µL of spent medium of each strain and 100 µL of cell suspension (30·10^3^ cells cm^-2^) adjusted to the given proportions and cell densities of the pre-cultures. Over a period of 5 days, gamete formation and initial cell formation was evaluated.

*Statistical Evaluation*

All data are depicted as mean ± standard error of the mean. The statistical analysis was done with square root transformed data in the open-source statistical program R v.4.0.3. (Team 2016). After testing for normal distribution and equal variances, effects were evaluated by applying *One-Way* *ANOVA* (α = 0.05) and a subsequent *Tukey´s* multiple comparison post-hoc test. In case of the clustering experiment multivariate analysis was applied to consider two dependent outcome variables (pairs and clusters) using the package MANOVA.rm (Friedrich et al. 2018). A *Mann-Whitney* rank sum test was applied to compare two groups.

Results

*Designation of Mating Types in Cylindrotheca closterium.*

To detect characteristics of mate finding and searching behaviour in *Cylindrotheca closterium* compatible strains of mating type (mt) Cyc1 and Cyc2 (Vanormelingen et al. 2013) were crossed in different ratios (Fig. 1). The strong accumulation of Cyc1 cells around Cyc2 cells revealed the attracting nature of Cyc2 cells and leads to the assignment of Cyc2 to *mating type*^¯^ (mt^¯^). Cells of Cyc1, on the other hand, seemed to be more motile searching for the opposite partner and thus representing *mating type*^+^ (mt^+^). Pair formation as well as cell cluster formation of distinct mating types were recognized by the differing cell lengths of both mating types. Cell clusters consist of at least three times more cells of the dominant mt (Fig. 1a). Mt^+^ cells that were either smaller or bigger than mt^¯^ both showed clustering around mt^¯^ (Fig. S1) which confirms that searching behaviour is independent of cell size concerning mt^+^. Crosses with an equal number of both mating types (5:5) resulted in most successful mating (11.7·10^3^ total interactions cm^-2^ out of 15·10^3^ possible pairs) comprising formation of pairs, clusters and triplets (Fig. 1b). As the proportion changed towards one mating type total interactions were limited by the partner present in the lowest density and thus less interactions cm^-2^ were monitored. In crosses with a low mt^+^:mt^¯^ ratio cells mostly formed pairs (40‑60% of total interaction, Fig. 1c). Pair formation also dominated the crosses of equal mating type ratio, comprising almost two thirds of all interactions. With a descending proportion of mt^¯^ cells accumulations of mt^+^ cells around mt^¯^ cells occurred. Clusters were even most prevalent in crosses with a majority of mt^+^ cells. In the 9:1 cross, clusters dominated the culture comprising more than 85% of all total interactions (4.2·10^3^ clusters cm^-2^). Clusters were still detected in crosses with a low mt^+^:mt^¯^ ratio but in a comparably low amount (10‑30% of total interactions). Taken together, these data show that each mating type has a specific behaviour and a defined functional purpose that shapes and structures the mate finding process.

*Bioassay Reveals Chemical Mediated Attraction of mt^+^.*

We hypothesized that chemical signalling is involved in the mate finding process of *C. closterium*. To verify this hypothesis, mt^+^ cell attraction towards mt^¯^ exudates was assessed. Exudates could be extracted via solid phase extraction using methanol as an eluant. A chemotaxis assay comprised of capillaries filled with medium extracts embedded in agar was optimized to meet the requirements of using benthic algae instead of bacteria (Abe et al. 2017). Extracts diffuse from small capillaries that are placed on the bottom of a well (Fig. 2a). Careful optimization included the use of smaller capillaries (5 µL instead of 20 µL) that were placed vertically in the well. A foil, holding the capillaries ensured a stable set-up and was required to counteract the extreme variability in initial experiments (Fig. S2) and increased robustness of this delicate procedure dramatically.
Capillaries filled with mt‾ medium extracts led to an accumulation of mt^+^ cells around the capillary opening 60 min after inserting the capillary (Fig. 2b). Cell counting revealed almost three times more mt^+^ cells around target capillaries than around control capillaries. Controls contained extract of the same mating type and showed no activity since cell numbers around the capillaries did not increase over time (Fig. 2c). We therefore suggest that mt^+^ cells must have received a signal from the mt‾ medium extract within the capillary and accumulated at its highest concentration. As expected, extracts of MT‾ cultures (sexually immature; >SST) did not trigger a response of mt^+^ cells.

Further, mt^+^ cells also responded to mt‾ medium extract by changing their motility behaviour. One hour after exposure to mt‾ medium extract, mt^+^ cells employed chemokinesis resulting in an increase of the overall mean speed (Fig. 3a). mt^+^ cells were 2.7 times faster compared to control cultures. Likewise, the travelled net distance of treated mt^+^ cells also increased (Fig 3b) which enhances mate finding. As expected, mt‾ cell motility was not affected by medium extract of mt^+^ (Fig 3b, c).

This leads to the assumption that a chemical cue is exuded by mt‾ cells as soon as they fall below the sexual size threshold (SST). The sexually mature cells start to constitutively produce this attraction pheromone without further induction. This pheromone plays an essential role in guiding mt^+^ cells and proves chemical involvement in the mate finding process of *C. closterium* to increase mating efficiency.

*Cell Cycle Regulation of mt^+^ during Mating.*

Besides cell attraction we investigated cell cycle regulations of *C. closterium* during mate finding. DNA content was measured with flow cytometry and reflected the cell cycle phases (G1, S+G2) of the cells. Initially, cultures of both mating types were successfully synchronized in their cell cycle by dark treatment as described by Vanormelingen et al. (2013). Cells were kept almost solely in G1 phase (>95%) of the cell cycle (Fig. 4a, top) and less than 5% of the cells entered the S+G2 phases of the cell cycle (Fig. 4b, black). As expected at least 20% of the cells proceeded into the S+G2 phases of the cell cycle 6 hrs after illumination (Fig. 4a, middle, Fig. 4b grey). A comparison of both mating types revealed that mt‾ spent medium caused an arrest of mt^+^ cells in G1 phase that lasted for at least 6 hrs (6 hrs, bottom). Surprisingly, mt‾ cultures treated with mt^+^ spent medium were not arrested but proceeded in the cell cycle. That contrasts the mt^+^ cultures that were arrested in G1 phase in the presence of mt‾ spent medium.
Overall, it confirms that a chemical cue is exuded by mt‾ cells and functions as a cell cycle regulation pheromone in *C.* *closterium*. The chemical signal is important upon the mating process only since medium of MT‾ cells (>SST, Fig. 4c) did not lead to an arrest of mt^+^ cells in the cell cycle. Thus, the signal responsible for the cell cycle arrest in mt^+^ is only produced by sexually mature mt‾ cells and is crucial for the sexual reproduction of *C. closterium*.

*Gametogenesis of mt^+^ is Triggered by Pheromone of mt*‾*.*

Lastly, we studied the mating process regarding gamete formation and subsequent initial cell formation over a period of 5 days (Fig. 5). Crossing cultures with a mt^+^:mt‾ ratio of either 1:9 or 9:1 resulted in equivalent numbers of initial cells. Compared to crosses with a more balanced mt ratio, these crosses were still efficient with respect to the numbers of compatible partners (Fig. S3). When mt‾ cells were dominant, gametes of each mating type developed equally on the first day. Their numbers decreased over time while compatible gametes paired effectively and completely evolved into initial cells. Almost no gametes of either mating type were found after 5 days (Fig. 5a). In crosses with a high mt^+^:mt‾ cell ratio not only mt‾ cells but the majority of mt^+^ cells developed into gametes. This led to an increase of mt^+^ gametes over time. Facing an insufficient number of compatible partners only few gametes developed into initial cells (Fig. 5b). Again, we observed mt^+^ cell clusters around mt‾ cells that resulted in an enhanced number of gametes. Gametes were only formed within those clusters (Vanormelingen et al. 2013). However, mt^+^ cells within the cluster, but with no direct contact to mt‾ cells also evolved into gametes. Non-pairing mt^+^ cells were unaffected. This suggests that mt‾ cells exude another short living instable signal that induces gametogenesis in mt^+^ cells. Based on this experiment we suggest a third pheromone to be involved in the sexual reproduction of *C. closterium* and proved that not only mate finding but sexual reproduction itself is chemically mediated.

Discussion

We investigated the heterothallic mating system of the benthic pennate diatom *Cylindrotheca closterium* with respect to its distinct mating types (mt) and the pheromones that are involved in the process of mate finding. *C. closterium* has mating types of opposite sexes Cyc1 and Cyc2 (Vanormelingen et al. 2013) that are morphologically indistinguishable. We showed that each mt, when it falls below the sexual size threshold (SST) fulfils a specific role, either to attract the partner (mt^¯^) or to purposely search for it (mt^+^). This suggests that mating behaviour in *C. closterium*, is genetically predetermined. It resembles the mating system of *Seminavis robusta* (Vanstechelman et al. 2013), which also constitutes of an attracting partner and a more motile searching one as soon as the SST is reached (Gillard et al. 2013). In contrast, in *Pseudo-nitzschia multistriata* the behaviour of mating types was interpreted as being under the control of cell size (Scalco et al. 2016) since the larger strain, independently of the mating type, actively searches for the smaller, less motile strain. For heterothallic diatom species mating types are not categorized in ‘+’ and ‘**_¯_**‘, *e.g. Haslea ostrearia*, or *Pseudo-nitzschia multistriata*. (D'Alelio et al. 2009; Davidovich et al. 2009) and their gametes are morphologically indistinguishable. However, assignment of male and female gametes is described frequently in araphid pennate diatoms, *e.g*. *Tabularia fasciculate and Pseudostaurosira trainorii*. Female gametes stay attached to the parental valves whereas the more motile male gametes facilitate contact with the females (Davidovich et al. 2010; Sato et al. 2011).

Notably, behaviour of mt^+^ cells in *C. closterium* is pheromone guided. A signal released by mt^¯^ cells enables mate finding like in *S. robusta* (Gillard et al. 2013). The attraction *bead assay* used for *S. robusta* could not be employed since cells attached to the unloaded beads as well (Gillard et al. 2013). We therefore established a novel capillary assay in which mt^+^ cells were attracted to extracts of mt^¯^ embedded in agar. A long response time of mt^+^ cells resulted, most likely, from an incomplete diffusion of chemical signals from the capillary. We excluded a random encountering like it was shown in *P.  trainorii*. Male gametes of *P.  trainorii* only head directly towards female gametes after an initial “random walk” to come within a close range of opposite sex (Sato et al. 2011). In *C. closterium*, mt^+^ cells response chemokinetically towards mt^¯^ exudates with respect to their pace. When mt^+^ cells sense the pheromone they move almost three times quicker. In *S. robusta* natural pairing only occurs after 6 hrs (Chepurnov et al. 2008) but directed attraction was proven (Bondoc et al. 2016). A similar analysis of directed movement is crucial to determine whether chemotaxis is occurring in *C. closterium* as well.

Other than in *S. robusta* or *P. trainorii* the pheromone production in *C. closterium* is only dependent on cell size. As soon as mt^¯^ cells get sexualized they secrete the pheromone and unconditioned mt^+^ cells are able to perceive the signal. In contrast, a reciprocal stimulation is known for *P. trainorii* where three different pheromones are involved in the sexual reproduction (Sato et al. 2011). The production of the attraction pheromone l‑Diprolin in *S. robusta* is also induced by a sex inducing pheromone (SIP) of mt^+^. Simultaneously, mt^¯^ produces SIP^¯^ that induces the development of a receptor in mt^+^ (Moeys et al. 2016).

Compared to *S. robusta* we confirm a rather short pre-mating induction phase in *C. closterium* (Vanormelingen et al. 2013). We observed a cell cycle arrest in mt^+^ cells induced by a cytostatic pheromone exuded by mt^¯^ similar to *S. robusta*. In *S. robusta* as well as *P. multistriata* both mating types are arrested in G1 of the cell cycle (Basu et al. 2017; Moeys et al. 2016). However, mt^¯^ cells in *P. multistriata* initiate the sexual process by undergoing meiosis earlier than mt^+^ whose response is slightly out of phase. Difference in cell cycle progression of mating types is also known in *S. robusta*. G1 synchronized mt^+^ cells progress in the cell cycle already 3h after light treatment whereas mt^¯^ only enter the G2/M phase after 9h (Bilcke et al. 2020). The mt^¯^ cells in *C. closterium* further promote gametogenesis of mt^+^ cells by producing another pheromone. Involvement of pheromones not only in the mate finding process but in the sexual reproduction itself is known for other pennate diatoms as well. In *P. trainorii* also female cells secrete a pheromone responsible for the sexual stimulation of male cells (Sato et al. 2011) and in *T.  fasciculata* gametogenesis is induced without cell contact (Davidovich et al. 2010).

Overall, the mating type behaviour and pheromone system of *C. closterium* has a unique complexity but includes aspects observed in other pennate diatoms like *S. robusta* and *P.* *trainorii*. Future experiments will focus on a metabolomics approach to chemically investigate the sexual phases of *C. closterium* in more detail and identify the attraction pheromone as well as the cytostatic pheromone (Fiorini et al. 2020; Gillard et al. 2013).

References

Abe T, Nakamura S, Kudo S (2017) Bioconvection induced by bacterial chemotaxis in a capillary assay. Biochem Biophys Res Commun 483:277-282 doi:<https://doi.org/10.1016/j.bbrc.2016.12.152>

Amato A (2010) Diatom Reproductive Biology: Living in a Crystal Cage. J Plant Reprod Biol 2:1-10

Apoya-Horton MD, Yin L, Underwood GJC, Gretz MR (2006) Movement modalities and response to environmental changes of the mudflar diatom *Cylindrotheca closterium* (Bacillariophyceae). J Phycol 42:379-390 doi:10.1111/j.1529-8817.2006.00194.x

Armbrust EV (2009) The life of diatoms in the world's oceans. Nature 459:185 doi:10.1038/nature08057

Basu S et al. (2017) Finding a partner in the ocean: molecular and evolutionary bases of the response to sexual cues in a planktonic diatom. New Phytol 215:140-156 doi:10.1111/nph.14557

Benoiston A-S, Ibarbalz FM, Bittner L, Guidi L, Jahn O, Dutkiewicz S, Bowler C (2017) The evolution of diatoms and their biogeochemical functions. Philos Trans R Soc Lond B Biol Sci 372:20160397 doi:10.1098/rstb.2016.0397

Bilcke G et al. (2020) Mating type specific transcriptomic response to sex inducing pheromone in the pennate diatom *Seminavis robusta*. ISME J doi:10.1101/2020.03.16.987719

Bondoc KGV, Lembke C, Lang SN, Germerodt S, Schuster S, Vyverman W, Pohnert G (2019) Decision-making of the benthic diatom *Seminavis robusta* searching for inorganic nutrients and pheromones. ISME J 13:537-546 doi:10.1038/s41396-018-0299-2

Bondoc KGV, Lembke C, Vyverman W, Pohnert G (2016) Searching for a Mate: Pheromone-Directed Movement of the Benthic Diatom *Seminavis robusta*. Microb Ecol 72:287-294 doi:10.1007/s00248-016-0796-7

Bonneure E, De Baets A, De Decker S, Van den Berge K, Clement L, Vyverman W, Mangelinckx S (2021) Altering the Sex Pheromone Cyclo(L-Pro-L-Pro) of the Diatom *Seminavis robusta* towards a Chemical Probe. Int J Mol Sci 22:1037

Chepurnov V, Mann D, Sabbe K, Vyverman W (2004) Experimental Studies on Sexual Reproduction in Diatoms. Int Rev Cytol 237:91-154 doi:10.1016/S0074-7696(04)37003-8

Chepurnov VA et al. (2005) Sexual reproduction, mating system, chloroplast dynamics and abrupt cell size reduction in *Pseudo-nitzschia pungens* from the North Sea (Bacillariophyta). Eur J Phycol 40:379-395 doi:10.1080/09670260500254735

Chepurnov VA et al. (2008) In search of new tractable diatoms for experimental biology Bioessays 30:692-702 doi:10.1002/bies.20773

Chepurnov VA, Mann DG, Vyverman W, Sabbe K, Danielidis DB (2002) Sexual Reproduction, mating system, and protoplast dynamics of *Seminavis* (Bacillariophyceae). J Phycol 38:1004-1019 doi:10.1046/j.1529-8817.2002.t01-1-01233.x

D'Alelio D, Amato A, Luedeking A, Montresor M (2009) Sexual and vegetative phases in the planktonic diatom *Pseudo-nitzschia multistriata*. Harmful Algae 8 doi:10.1016/j.hal.2008.05.004

Davidovich AN (2001) Species specific sizes and size range of sexual reproduction in diatoms. Proc. 16th Int. Diatom Symp. (Athens and Aegean Islands, Aug. 25–Sept. 1, 2000), Athens: Univ. Athens

Davidovich AN, Kaczmarska I, Ehrman MJ (2010) Heterothallic and homothallic sexual reproduction in *Tabularia fasciculata* (Bacillariophyta). Fottea 10:251-266 doi:10.5507/fot.2010.016

Davidovich NA, Mouget J-L, Gaudin P (2009) Heterothallism in the pennate diatom *Haslea ostrearia* (Bacillariophyta). Eur J Phycol 44:251-261 doi:10.1080/09670260802710301

Falkowski PG, Barber RT, Smetacek V (1998) Biogeochemical Controls and Feedbacks on Ocean Primary Production. Science 281:200 doi:10.1126/science.281.5374.200

Fiorini F, Borgonuovo C, Ferrante MI, Brönstrup M (2020) A Metabolomics Exploration of the Sexual Phase in the Marine Diatom *Pseudo-nitzschia multistriata*. Mar Drugs 18:313 doi:10.3390/md18060313

Frenkel J, Vyverman W, Pohnert G (2014) Pheromone signaling during sexual reproduction in algae Plant J 79:632-644 doi:10.1111/tpj.12496

Friedrich S, Konietschke F, Pauly M (2018) Analysis of Multivariate Data and Repeated Measures Designs with the R Package MANOVA.RM. New York

Gillard J et al. (2013) Metabolomics Enables the Structure Elucidation of a Diatom Sex Pheromone. Angew Chem Int Ed 52:854-857 doi:<https://doi.org/10.1002/anie.201208175>

Guillard RRL (1975) Culture of Phytoplankton for Feeding Marine Invertebrates. In: Smith WL, Chanley MH (eds) Culture of Marine Invertebrate Animals: Proceedings — 1st Conference on Culture of Marine Invertebrate Animals Greenport. Springer US, Boston, MA, pp 29-60. doi:10.1007/978-1-4615-8714-9_3

Lewis WM (1987) The cost of sex. In: Stearns S.C. (eds) The Evolution of Sex and its Consequences. Experienta Supplementum, vol 55. Birkhäuser, Basel,

Malviya S et al. (2016) Insights into global diatom distribution and diversity in the world’s ocean Proc Nat Acad Sci 113:E1516 doi:10.1073/pnas.1509523113

Moeys S et al. (2016) A sex-inducing pheromone triggers cell cycle arrest and mate attraction in the diatom *Seminavis robusta*. Sci Rep 6:19252 doi:10.1038/srep19252

Najdek M, Blažina M, Djakovac T, Kraus R (2005) The role of the diatom *Cylindrotheca closterium* in a mucilage event in the northern Adriatic Sea: coupling with high salinity water intrusions. J Plankton Res 27:851-862 doi:10.1093/plankt/fbi057

Nakov T, Beaulieu JM, Alverson AJ (2018) Accelerated diversification is related to life history and locomotion in a hyperdiverse lineage of microbial eukaryotes (Diatoms, Bacillariophyta). New Phytol 219:462-473 doi:<https://doi.org/10.1111/nph.15137>

Osuna-Cruz CM et al. (2020) The *Seminavis robusta* genome provides insights into the evolutionary adaptations of benthic diatoms. Nat Commun 11:3320 doi:10.1038/s41467-020-17191-8

Poulíčková A, Mann DG (2019) Diatom Sexual Reproduction and Life Cycles. In: Diatoms: Fundamentals and Applications. pp 245-272. doi:10.1002/9781119370741.ch11

Russo MT et al. (2018) MRP3 is a sex determining gene in the diatom *Pseudo-nitzschia multistriata*. Nat Commun 9:5050-5050 doi:10.1038/s41467-018-07496-0

Sato S, Beakes G, Idei M, Nagumo T, Mann DG (2011) Novel Sex Cells and Evidence for Sex Pheromones in Diatoms. PLOS ONE 6:e26923 doi:10.1371/journal.pone.0026923

Scalco E, Amato A, Ferrante MI, Montresor M (2016) The sexual phase of the diatom *Pseudo-nitzschia multistriata*: cytological and time-lapse cinematography characterization. Protoplasma 253:1421-1431 doi:10.1007/s00709-015-0891-5

Scalco E, Stec K, Iudicone D, Ferrante MI, Montresor M (2014) The dynamics of sexual phase in the marine diatom *Pseudo-nitzschia multistriata* (Bacillariophyceae). J Phycol 50:817-828 doi:doi:10.1111/jpy.12225

Smetacek V (1998) Diatoms and the silicate factor. Nature 391:224-225 doi:10.1038/34528

Stal LJ, Bolhuis H, Cretoiu MS (2019) Phototrophic marine benthic microbiomes: the ecophysiology of these biological entities. Environ Microbiol 21:1529-1551 doi:10.1111/1462-2920.14494

Stock W, Vanelslander B, Rüdiger F, Sabbe K, Vyverman W, Karsten U (2019) Thermal Niche Differentiation in the Benthic Diatom *Cylindrotheca closterium* (Bacillariophyceae) Complex Frontiers in Microbiology 10 doi:10.3389/fmicb.2019.01395

Team RC (2016) R: A Language and Environment for Statistical Computing. vol 1. Vienna, Austria. doi:10.1890/0012-9658(2002)083[3097:CFHIWS]2.0.CO;2

Vanormelingen P, Vanelslander B, Sato S, Gillard J, Trobajo R, Sabbe K, Vyverman W (2013) Heterothallic sexual reproduction in the model diatom *Cylindrotheca*. Eur J Phycol 48:93-105 doi:10.1080/09670262.2013.772242

Vanstechelman I, Sabbe K, Vyverman W, Vanormelingen P, Vuylsteke M (2013) Linkage Mapping Identifies the Sex Determining Region as a Single Locus in the Pennate Diatom *Seminavis robusta*. PLOS ONE 8:e60132 doi:10.1371/journal.pone.0060132

von Quillfeldt CH, Ambrose WG, Clough LM (2003) High number of diatom species in first-year ice from the Chukchi Sea. Polar Biol 26:806-818 doi:10.1007/s00300-003-0549-1

Yallop ML, de Winder B, Paterson DM, Stal LJ (1994) Comparative structure, primary production and biogenic stabilization of cohesive and non-cohesive marine sediments inhabited by microphytobenthos. Estuarine, Coastal and Shelf Science 39:565-582 doi:<https://doi.org/10.1016/S0272-7714(06)80010-7>

Figure Legends:

**Fig. 1 Mating type determination in *Cylindrotheca closterium*.** Crosses of Cyc1 (C2) and Cyc2 (CA1.15) in different mating type ratios 5 h after mating, leading to the identification of the respective mating type. (a) Inverted microscope images of the crosses showing an increase of clusters (blue, at least 3:1 cells of the prevailing strain) when Cyc1 cells become prevalent. Most paired cells (orange) can be seen when Cyc1 and Cyc2 are present in similar numbers. Scale = 100 µm (b) Means ± s.e. (n = 3) of total sexual events, (c) clusters (blue bars), and pairs (orange bars) depending on different mating type ratios. Significance was calculated with a *one-way* ANOVA (b) or a multivariate ANOVA (c) (α = 0.05, *Tukey’s* multiple comparison post-hoc test). Letters a, b, c, d and e and letters A, B, C, and D represent statistically significantly different groups (p < 0.001).

**Fig. 2 Chemical attraction of mt^+^ cells towards mt^¯^ cells in *C. closterium*.** In a chemoattraction assay glass capillaries were filled with different medium extracts embedded in agar to monitor mt^+^ cell accumulation around it. (a) Scheme of capillary assay. (b) Capillary filled with mt^¯^ medium extract in agar is depicted (picture is taken from below the set-up) at the start of the experiment (left). mt^+^ cells accumulated around the capillary after 60 min (right). Pictures were grey scale adjusted. Scale = 200 µm
(c) Comparison of mt^+^ cell accumulation (t_60min_/t_0min_) around capillaries filled with medium extract of mt^+^ (control) and of mt^¯^ < SST (mt^¯^) and > SST (MT^¯^), n = 4. Significance was calculated with *one-way* ANOVA (α = 0.05, *Tukey* test). Letters a and b represent significantly different groups (p < 0.001).

**Fig. 3 Chemokinesis in *C. closterium*.** In a motility assay cells were exposed to medium extract of the opposite mating type (n = 4). (a) The overall mean speed of mt^+^ cells in the treatment is almost three times higher than in the control. (b) Displacement analysis of treated mt^+^ cells showed a significantly higher travelled net distance in 30 s. (c,d) Treated mt‾ cells did not change regarding their mean speed or the distance they travelled compared to the control. Significance was calculated with a *Mann-Whitney* rank sum test (*p* < 0.001).

**Fig. 4** **Cell cycle arrest of mt^+^ cells of *C. closterium* in G1 phase measured by flow cytometry.** Cells were dark‑synchronized in G1 phase (G2 < 5%) and treated with spent medium of the opposite mating type. (a) DNA histograms of mt^+^ cells showing a synchronized culture (top), a control culture (middle) and conditioned culture (bottom) 6 hours after illumination. (b) Comparison of mt^+^ treated with mt^¯^ spent medium and vice versa. Treated mt^+^ cells are arrested in G1 phase after 6 hours. (c) mt^+^ cells are only arrested by spent medium from mt- (below SST) not by spent medium of larger MT^¯^ cells. Means (n = 3,4) of proportion of cells in G2 phase at t = 0 hrs (black) and t = 6 hrs after illumination (grey) are depicted. Significance was calculated with a *one-way* ANOVA (α = 0.05, *Tukey* test). Letters a and b represent groups of non-significantly different distribution (p < 0.0001).

F**ig. 5** **Gametogenesis of mt^+^ cells**. Crossing mt^+^ and mt‾ of *C. closterium* (n = 3) resulted in gamete (bars) and initial cell (dots) formation, which were monitored for 5 days. (a) A mt^+^: mt^¯^ ratio of 1:9 showed a decrease of gametes among the increase of initial cells, whereas (b) a mating type ratio of 9:1 showed high numbers of gametes.
